# Effects of FMEA-based bleeding risk-stratified nursing on recovery process and safety after coronary angiography

**DOI:** 10.3389/fpubh.2026.1964833

**Published:** 2026-09-25

**Authors:** Xiaoping Guo, Lingxia Fan, Yanzhuo Ma, Wei Li

**Affiliations:** Department of Cardiology, Bethune International Peace Hospital, Shijiazhang, China

**Keywords:** bleeding risk-stratified nursing, coronary angiography, failure mode and effects analysis, pain management, postoperative recovery trajectory

## Abstract

**Background:**

Access-site bleeding and postprocedural pain after coronary angiography are linked by a “bleeding–inflammation–pain” positive feedback coupling mechanism. Traditional event-driven nursing models lack prospective risk stratification and preemptive control, making it difficult to break this vicious cycle at its source.

**Purpose:**

To systematically evaluate the effects of a Failure Mode and Effects Analysis (FMEA)–based bleeding risk-stratified nursing intervention on recovery process and safety outcomes in patients after coronary angiography.

**Methods:**

A prospective randomized controlled study was conducted in a tertiary teaching hospital from January 2023 to December 2024. A total of 240 patients scheduled for elective coronary angiography or angiography with interventional therapy were enrolled and allocated to a control group or an FMEA group. In the FMEA group, failure modes in the nursing workflow were systematically identified, the Risk Priority Number (RPN) was calculated, and patients were stratified into low-, moderate-, and high-risk tiers, with correspondingly differentiated nursing protocols, low-risk patients received routine monitoring and standard haemostasis; moderate-risk patients received intensified monitoring, prolonged compression, and structured activity guidance; and high-risk patients received continuous monitoring, individualised analgesic titration, and nurse-led progressive ambulation training. Primary outcomes included postoperative pain intensity, access-site bleeding volume, and hemoglobin decline; secondary outcomes were time to first ambulation and functional independence score at discharge; system-level indicators included unplanned nursing interventions, nursing documentation time, and safety incident reporting rate.

**Results:**

Compared with the control group, the FMEA group showed significantly lower NRS pain scores at 24 h postoperatively and a 23.4% reduction in cumulative opioid consumption at 72 h. The incidence of clinically significant access-site bleeding decreased from 17.5 to 8.3%, estimated 48-h bleeding volume was reduced by 22.9%, and postoperative hemoglobin decline was significantly less in the FMEA group. First ambulation time was advanced by approximately 8.2 h, functional independence score at discharge improved by 11.2%, and length of hospital stay was shortened by about 1.8 days, with no significant difference in 30-day readmission rates between groups. At the system level, the FMEA group had significantly fewer unplanned nursing interventions, shorter documentation time, and a lower safety incident reporting rate.

**Conclusion:**

This single-centre prospective controlled study suggests that FMEA-based bleeding risk-stratified nursing was associated with improvements in short-term bleeding-related, pain-related, mobility-related, and selected workflow outcomes. These findings support the potential of the FMEA framework to facilitate the transition from an event-driven, reactive nursing model to a risk-driven, preemptive approach. However, given the exploratory nature of this study, the results should be interpreted as preliminary and require further validation.

## 1 Introduction

Coronary angiography remains the gold standard for the diagnosis and interventional treatment of cardiovascular disease. However, the quality of postoperative recovery has long been constrained by two core interrelated problems: access-site bleeding and postoperative pain, which together form a vicious cycle (1). Access-site bleeding is not only the most common local vascular complication after coronary angiography but also a substantially underestimated trigger of systemic stress responses (2, 3). Bleeding itself directly activates nociceptors through local haematoma formation, increased tissue tension, and pressure-induced ischaemia, thereby producing acute pain. Concurrently, extravasated erythrocyte degradation products and damage-associated molecular patterns (DAMPs) released from injured tissues elicit a local sterile inflammatory response, promoting the release of prostaglandin E₂ (PGE₂), bradykinin, and multiple pro-inflammatory cytokines, which further lower pain thresholds and amplify pain signals (4, 5). This “bleeding–inflammation–pain” positive feedback coupling mechanism transforms pain from a simple traumatic response to puncture into a complex inflammatory hyperalgesic state, substantially increasing opioid analgesic requirements (6). Moreover, pain-driven protective immobilisation further delays ambulation and functional recovery, while delayed mobilisation impairs local venous return and haematoma resolution, thus perpetuating a closed-loop vicious cycle: bleeding → pain → immobilisation → delayed recovery → persistent bleeding risk (7). Despite this, systematic recognition of this vicious cycle in current clinical nursing practice remains inadequate; most interventions target only a single component, lacking an integrated strategy to interrupt the cycle at its source.

To address these issues, the conventional nursing model widely adopted in clinical practice is predominantly event-driven, meaning that nursing actions are initiated only after symptoms or adverse events have occurred, haemostasis is reinforced after bleeding is observed, analgesia is administered after pain is reported, and rehabilitation guidance is offered after mobility becomes restricted (8, 9). Although this reactive approach may be effective in managing individual incidents, it has fundamental limitations: (1) temporal lag: there is an unavoidable time window between risk formation, event occurrence, and nursing response, during which pathophysiological cascades have already been set in motion; (2) lack of risk stratification: all patients receive similar intensity of routine care, leaving high-risk individuals without adequate early warning and intensified monitoring, while low-risk patients may receive unnecessary nursing intensity; and (3) fragmented care decisions: bleeding management, pain control, and rehabilitation guidance are handled as separate nursing components without systematic integration based on a shared underlying risk mechanism. This disconnect between resource allocation and actual patient risk levels impedes the achievement of an optimal balance between clinical outcomes and healthcare costs at the population level.

The root of these limitations lies in the absence of a prospective, quantitative risk-assessment tool to guide nursing decisions. Failure Mode and Effects Analysis (FMEA), a proactive system reliability tool originating from the aerospace and nuclear industries, has been progressively introduced into healthcare risk management and quality improvement over the past two decades (10, 11). The core philosophy of FMEA is not “damage control after the event” but “prevention before harm occurs”: it systematically deconstructs each critical step in a clinical process, identifies potential failure modes, analyses their root causes and clinical consequences, and thereby enables numerical prioritisation of risks (12, 13). In essence, this approach brings diverse and otherwise incomparable risks into a unified quantitative framework, shifting nursing decisions from experience-based to data-driven. Within nursing risk management, the introduction of FMEA implies a transition from “uniform protocol execution” to “differentiated intervention based on individualised risk assessment”: it equips nursing teams with the ability to prospectively stratify patients’ bleeding risk upon admission and to proactively tailor monitoring frequency, compression haemostasis protocols, analgesic strategies, and activity guidance intensity before risks materialise into adverse events (14). Nevertheless, despite its extensive validation in medication safety, infection control, and surgical safety checklists, the systematic application of FMEA to bleeding-stratified nursing after coronary angiography remains unexplored. Whether this quantified risk-grading approach can effectively break the “bleeding–pain–immobilisation” cycle and systematically optimise recovery trajectories has not yet been supported by clinical evidence (15).

Although existing studies have focused on single-domain bleeding risk assessments or isolated improvements in individual nursing procedures, current routine nursing practice still faces two key limitations: a disconnect between risk assessment and differentiated care decisions, and fragmented interventions that fail to address the interrelated “bleeding–pain–immobilisation–delayed recovery” chain as an integrated whole (16). The FMEA framework, by integrating risk quantification, workflow failure mode identification, and prospective stratified intervention, precisely fills this translational gap (10). At the system level, it serves as a process-improvement tool that identifies workflow vulnerabilities and optimises protocol design; at the individual level, it converts system-level risk factors into patient-specific risk stratification via RPN quantification, enabling precise matching of nursing intensity to individual risk (17). We hypothesise that, compared with routine care, this FMEA-based nursing strategy will improve pain control and bleeding management, accelerate functional recovery, and shorten hospital stay without increasing 30-day readmission risk (18). Pain intensity and bleeding-related indicators were prespecified as co-primary outcomes favouring the FMEA group, while functional recovery and system-level safety indicators were designated as secondary or exploratory endpoints (19).

Given these research gaps, we designed and conducted a prospective controlled study to systematically evaluate the effects of an FMEA-based bleeding risk-stratified nursing intervention on recovery process and safety in patients after coronary angiography. Specifically, we mapped the nursing workflow to identify key failure modes related to postoperative bleeding, used RPN scoring to establish individualised three-tier risk stratification, and assigned differentiated nursing intensity accordingly. The clinical value of this model was comprehensively assessed across four dimensions: pain control, bleeding management, functional recovery, and system-level safety and resource utilisation. The findings are expected to provide evidence supporting the transformation of interventional nursing from an event-driven, reactive paradigm to a risk-driven, precision preventive paradigm.

## 2 Methods

### 2.1 Study design and participants

This was a prospective randomized controlled trial conducted at a single tertiary teaching hospital between January 2023 and December 2024. Patients in both groups were cared for by the same team of cardiologists and nursing staff to minimise the impact of personnel differences on outcomes. Inclusion criteria were: (1) age 18–75 years; (2) scheduled for elective coronary angiography or coronary angiography with interventional therapy; (3) expected postoperative inpatient observation ≥5 days; and (4) American Society of Anesthesiologists (ASA) physical status I–III. Exclusion criteria were: (1) coexisting coagulation dysfunction; (2) dependence on long-acting anticoagulants that could not be adjusted perioperatively; (3) severe cognitive impairment precluding cooperation with pain assessment; and (4) emergency coronary angiography.

A total of 240 patients were enrolled and randomly assigned in a 1:1 ratio to either a control group (routine care, *n* = 120) or an FMEA group (FMEA-based bleeding risk-stratified nursing, *n* = 120) (20). The random sequence was generated by a statistician not involved in clinical care or nursing, using a random number table. To ensure allocation concealment, assignment codes were placed in sequentially numbered, opaque, sealed envelopes. Eligibility screening and informed consent were conducted by trained research nurses who were not responsible for group assignment. After enrolment, a separate nursing quality-control staff member, who was blinded to patient baseline data and not participating in outcome assessments, opened the envelopes in sequence to assign participants to either group. All patients completed the assigned nursing protocols and follow-up, with no dropouts.

The sample size was not derived from a conventional power calculation, as this was an exploratory quality improvement study rather than a confirmatory randomised controlled trial. The total of 240 patients was determined based on the centre’s average monthly caseload of comparable procedures (approximately 10 cases per month), the 24-month recruitment window, and practical feasibility, while ensuring that the final sample was adequate to maintain baseline balance and support exploratory analyses.

### 2.2 Ethical considerations

This study was conducted in accordance with the principles of the Declaration of Helsinki. The research protocol was approved by the Ethics Committee of Bethune International Peace Hospital (Approval No.: 2025-KY-88, Date: 2025.4.29). Written informed consent was obtained from all patients prior to their participation. Patients were assured of their right to withdraw from the study at any time without affecting their subsequent treatment. This study is a single-centre nursing process improvement project without drug or device interventions, and its protocol does not involve risks beyond routine care. Therefore, prospective clinical trial registration was not required under China’s Measures for Ethical Review of Biomedical Research Involving Humans and relevant registration guidelines. Nevertheless, the full study protocol was archived as part of the ethics approval dossier. The study protocol was submitted to the hospital ethics committee for initial review at the end of 2022, and oral approval was obtained before the study commenced in January 2023. The formal written approval document was delayed due to internal restructuring and leadership transitions within the ethics committee during 2023–2024, and was finally issued on April 29, 2025. Therefore, April 29, 2025, is the formal issuance date of the approval document, not the date when the ethics review was actually approved; the ethical review had in fact been completed before the study started.

### 2.3 Intervention protocol

Patients in the control group received standard postoperative care after coronary angiography, including routine vital-sign monitoring, conventional observation of the access site, on-demand analgesia, and standardised discharge instructions.

Patients in the FMEA group received FMEA-based postoperative bleeding risk-stratified nursing. The intervention was implemented by a specially trained cardiovascular interventional nursing team comprising one senior interventional nurse (≥10 years of experience), one cardiovascular specialist nurse, and one hospital quality management officer. All team members received unified training in FMEA methodology prior to study initiation, covering failure mode identification, severity (S), occurrence (O), and detectability (D) scoring criteria, RPN calculation rules, and the standardised implementation of differentiated nursing protocols.

The implementation process is illustrated in Figure 1. First, the team performed a structured mapping of the nursing workflow and identified four key failure modes related to post-angiography bleeding: delayed access-site inspection; inadequate compression haemostasis and monitoring; suboptimal perioperative anticoagulation coordination; and insufficient patient education on activity restriction and bleeding warning signs. Second, for each failure mode, S, O, and D were scored on a 1–10 scale using a team consensus approach: the three members independently assigned scores, followed by collective discussion until consensus was reached, to eliminate individual bias. The Risk Priority Number (RPN) was calculated as RPN = S × O × D. Third, based on RPN thresholds, patients were classified into three risk tiers: low risk (RPN < 80), moderate risk (80 ≤ RPN ≤ 160), and high risk (RPN > 160). These thresholds were derived from widely applied empirical risk classification rules in classical FMEA literature and were further validated by a retrospective internal calibration using bleeding event data from 100 patients who underwent similar procedures at our centre prior to this study.

**Figure 1:**
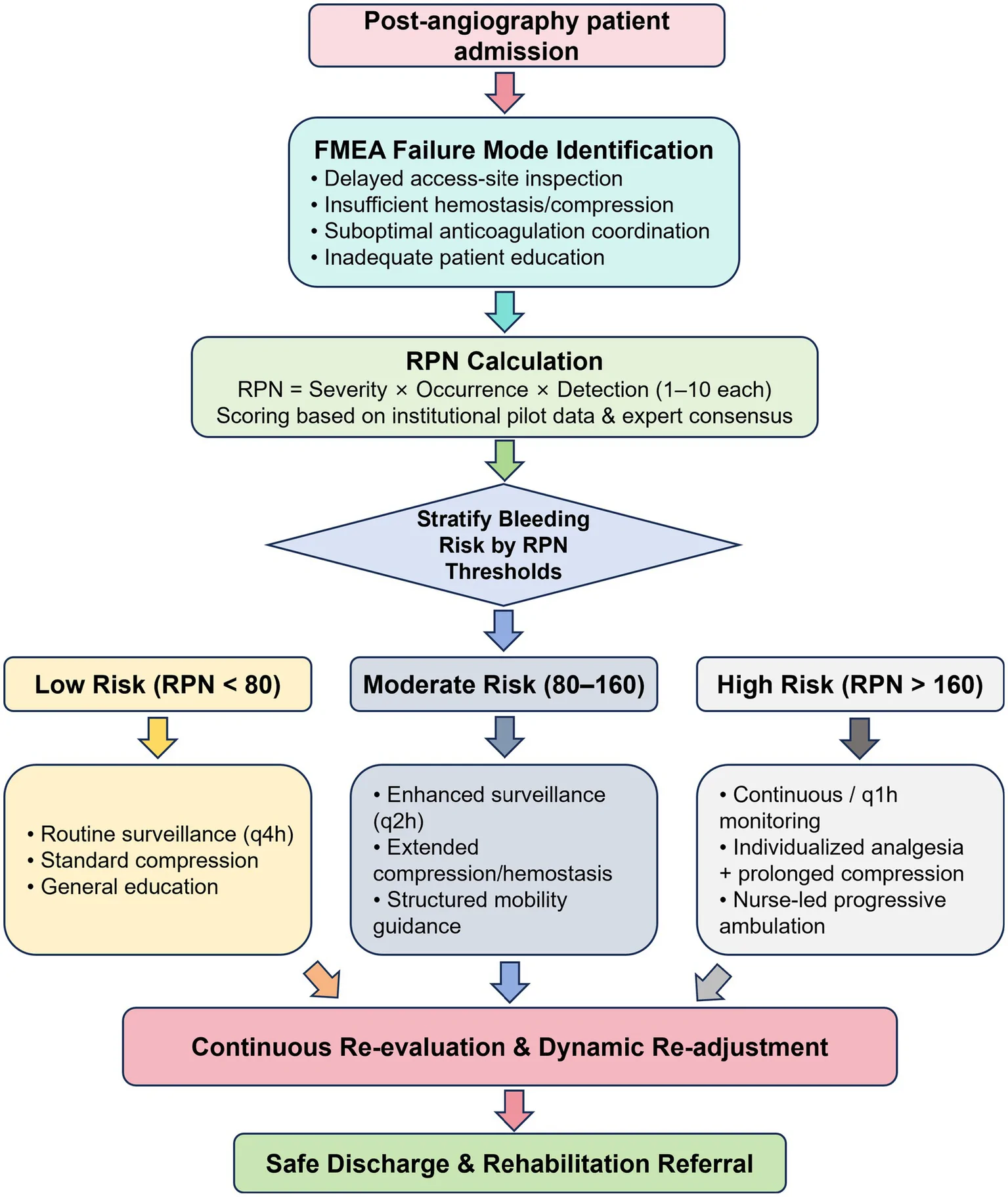
Flowchart of the FMEA-based postoperative bleeding risk-stratified nursing implementation process.

Finally, differentiated nursing intensity was assigned accordingly: low-risk patients received routine monitoring (q4h), standard compression haemostasis, and general education; moderate-risk patients received intensified monitoring (q2h), prolonged compression, and structured activity guidance; high-risk patients received continuous or q1h monitoring, individualised analgesic titration combined with prolonged compression, and nurse-led progressive ambulation training. Throughout the nursing process, RPN was dynamically reassessed and the intervention level was adjusted based on changes in the patient’s clinical status, serving as the escalation and referral mechanism.

### 2.4 Outcome measures

Primary outcomes included: (1) pain intensity at 12 h, 24 h, 48 h, and 72 h postoperatively, assessed using the Numerical Rating Scale (NRS, 0–10); (2) estimated access-site bleeding volume at 24 h and 48 h postoperatively; and (3) difference in haemoglobin between pre-procedure and postoperative day 3.

For bleeding-related outcomes, clinically significant access-site bleeding was defined as access-site bleeding requiring additional haemostatic intervention, prolonged compression, or resulting in a haemoglobin decline of ≥1.5 g/dL. Severe bleeding was defined as a stricter subset of clinically significant bleeding, specifically bleeding requiring blood transfusion, surgical intervention, or leading to haemodynamic instability. Estimated bleeding volume was based on a comprehensive assessment combining the area of puncture-site dressing saturation, gauze weighing, and drainage volume, recorded by trained ward nurses during each puncture-site assessment. Haemoglobin was measured before the procedure and on postoperative day 3, with all samples collected in the morning under fasting conditions; pre-procedural haemoglobin was balanced between groups at baseline (Table 1) and included as a covariate in multivariable regression models. Standardised intraoperative and postoperative fluid protocols were applied identically in both groups.

**Table 1:** Baseline demographic, clinical, perioperative, and haematological characteristics of patients undergoing coronary angiography.

Variable	Control group (*n* = 120)	FMEA group (*n* = 120)	*p*-value
Age (years)	55.7 ± 11.2	56.3 ± 10.8	0.684
Male sex, *n* (%)	63 (52.5)	66 (55.0)	0.712
Body mass index (kg/m^2^)	24.8 ± 3.1	25.1 ± 3.4	0.493
Smoking history, *n* (%)	41 (34.2)	44 (36.7)	0.691
ASA physical status I–II, *n* (%)	89 (74.2)	92 (76.7)	0.657
Diagnostic coronary angiography, *n* (%)	68 (56.7)	71 (59.2)	0.694
Coronary angiography with interventional procedures, *n* (%)	52 (43.3)	49 (40.8)	0.694
Local or combined anesthesia, *n* (%)	97 (80.8)	100 (83.3)	0.615
Procedure duration (min)	134.6 ± 28.9	137.2 ± 30.1	0.548
Vascular access route, radial *n* (%)	105 (87.5)	107 (89.2)	0.682
Femoral *n* (%)	15 (12.5)	13 (10.8)	—
Pre-procedural hemoglobin (g/dL)	13.6 ± 1.4	13.5 ± 1.3	0.731
Platelet count (×10^9^/L)	236.4 ± 41.8	231.9 ± 39.6	0.402
International normalized ratio (INR)	1.02 ± 0.09	1.03 ± 0.08	0.558
Activated partial thromboplastin time (s)	31.4 ± 3.6	31.8 ± 3.4	0.447
Fibrinogen (g/L)	3.18 ± 0.54	3.21 ± 0.51	0.682
Serum albumin (g/L)	41.2 ± 4.8	40.7 ± 5.1	0.472
Estimated glomerular filtration rate (mL/min/1.73 m^2^)	86.5 ± 14.2	84.9 ± 13.7	0.389

Data source: Prospectively collected pre-procedural clinical records of patients undergoing coronary angiography (January 2023–December 2024).

For pain assessment, at each time point the resting NRS score was recorded. If the score was ≥4, analgesics were administered according to the physician’s order, and the NRS score was reassessed after administration to evaluate the analgesic effect. Cumulative opioid consumption was converted to morphine milligram equivalents (MME) using standard equianalgesic conversion factors based on the American Society for Pain Management Nursing (ASPMN) Opioid Conversion Guidelines and the WHO Analgesic Ladder principles.

Secondary outcomes included: (1) time to first assisted ambulation, defined as the first time the patient stood up or walked with assistance after surgery, with the time point recorded by nurses; and (2) Functional Independence Measure (FIM) score at discharge (including subscores for mobility, transfer ability, and self-care). FIM assessment was performed on the day of discharge by trained research nurses; the scale has been validated in the Chinese population (21, 22). “Successful mobilisation” was defined as achieving assisted ambulation within 24 h postoperatively, and “discharge readiness” was quantified using a patient-reported readiness-for-discharge score (range 0–10), completed by the patient on the day of discharge.

Safety and system-level indicators included: (1) incidence of opioid-related adverse events; (2) number of unplanned nursing interventions, unplanned access-site assessments, and proportion of emergency analgesic adjustments; and (3) postoperative monitoring hours, utilisation rate of high-dependency monitoring resources, night-shift escalation calls, nursing workload index, nursing documentation time, and safety incident reporting rate. For these indicators, “unplanned nursing interventions” were defined as nursing activities performed at unscheduled time points triggered by changes in patient condition or adverse events, extracted item-by-item from the nursing documentation system. The “nursing workload index” was calculated using a weighted scoring method based on nursing hours per patient day (NHPPD), integrating direct care time, indirect care time, and documentation time, converted into quantitative scores according to the standard weights of our hospital’s nursing department. “Safety incident reports” were defined as any safety events related to postoperative care recorded in the hospital’s adverse event reporting system, expressed as the number of reported events per 100 patients.

All outcome assessors, including those performing NRS pain scoring, FIM functional assessments, and bleeding evaluations, were not involved in group assignment and were blinded to participants’ group allocation. Group allocation information was not explicitly indicated in nursing records accessible to assessors. All data were extracted independently by two trained researchers to ensure data consistency.

### 2.5 Statistical analysis

Data were analysed using SPSS version 26.0. Continuous variables were tested for normality using the Kolmogorov–Smirnov test. Normally distributed data are presented as mean ± standard deviation and were compared between groups using independent-samples *t*-tests. Categorical variables are expressed as frequencies (percentages) and were compared using the chi-squared test or Fisher’s exact test as appropriate. For binary outcomes, effect estimates (risk differences) with 95% confidence intervals were derived from between-group comparisons and are presented alongside frequencies and *p*-values in the corresponding tables. Pain scores over time were compared between groups using repeated-measures analysis of variance. Time to first ambulation was analysed using Kaplan–Meier estimation with cumulative distribution curves. Correlations were assessed using Pearson’s correlation coefficient and linear regression. All tests were two-sided, and a *p*-value < 0.05 was considered statistically significant. Given the exploratory nature of this quality improvement study, all analyses were interpreted as hypothesis-generating rather than confirmatory, and no interim analyses were performed. Access route (radial vs. femoral) was included as a covariate in multivariable regression models to adjust for its potential confounding effects on the primary outcomes.

## 3 Results

### 3.1 Baseline data and between-group balance verification

A total of 240 patients undergoing coronary angiography were enrolled, with 120 in the control group and 120 in the FMEA group. All patients completed the assigned nursing protocols and follow-up, with no dropouts. The age distribution boxplots showed that the median ages and interquartile ranges were highly overlapping between the two groups, with mean ages of 55.7 years and 56.3 years, respectively (Figure 2A). BMI distribution boxplots indicated generally similar body habitus between groups, with only a few outlying values in both high and low ranges (Figure 2B). Probability density histograms of procedure duration demonstrated that both groups peaked at 120–150 min, with almost entirely overlapping distribution patterns (Figure 2C). Pre-procedural haemoglobin scatterplots further confirmed that baseline haemoglobin levels were comparable across the two groups. These visualisations collectively suggested that the groups were well matched in key demographic and clinical characteristics (Figure 2D).

**Figure 2:**
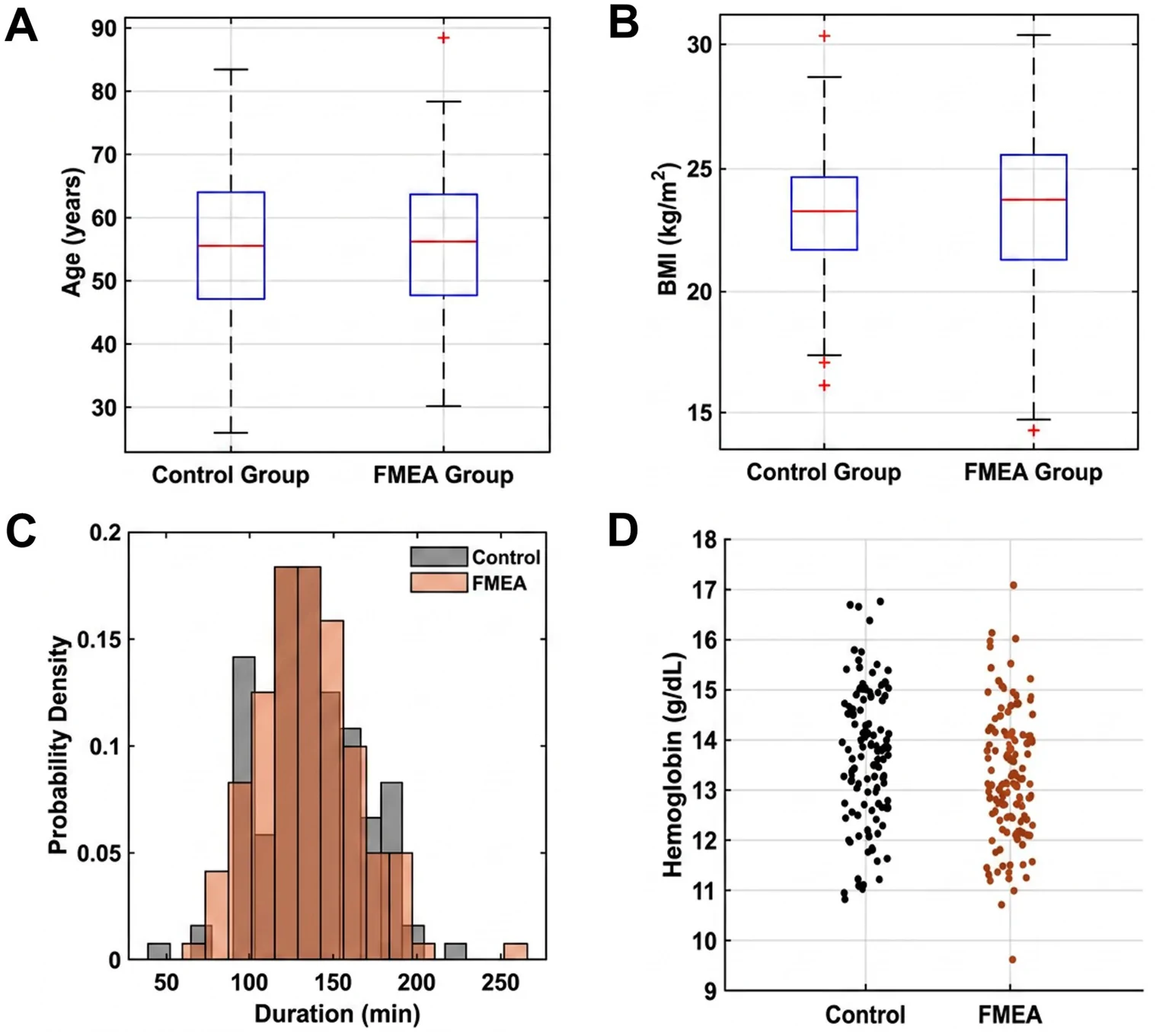
Comparison of baseline demographic and clinical characteristics between groups. **(A)** Boxplot of age distribution. **(B)** Boxplot of body mass index (BMI) distribution. **(C)** Probability density histogram of procedure duration. **(D)** Scatterplot of pre-procedural haemoglobin levels.

Table 1 presents the complete baseline demographic, clinical, perioperative, and haematological characteristics for both groups. Regarding demographic indices, age, proportion of males, BMI, and smoking history did not differ significantly between groups. The proportions of patients with ASA physical status I–II were 74.2 and 76.7%, respectively, indicating comparable baseline overall health status. For perioperative indices, the proportions of diagnostic coronary angiography, angiography with interventional procedures, and local or combined anaesthesia were evenly distributed between groups. Procedure durations were (134.6 ± 28.9) min and (137.2 ± 30.1) min, respectively, suggesting similar surgical complexity and trauma exposure. In haematological indices, pre-procedural haemoglobin, platelet count, international normalised ratio, activated partial thromboplastin time, fibrinogen, serum albumin, and estimated glomerular filtration rate, all related to coagulation and nutritional–metabolic status, showed no significant between-group differences. These results indicate that the control and FMEA groups were highly balanced with respect to age composition, sex distribution, body mass index, surgical complexity, anaesthesia type, and baseline coagulation function, key prognostic factors, so that subsequent differences in outcome measures can be attributed to the FMEA-based bleeding risk-stratified nursing intervention itself rather than to baseline confounding.

### 3.2 Bleeding precontrol effectiveness and pain/opioid reduction

Postoperative pain control is a core dimension for evaluating nursing intervention effectiveness. Figure 3A presents the trends in NRS pain scores from 12 to 72 h postoperatively; the FMEA group had significantly lower pain scores at all time points. Both groups showed a decreasing trend over time, but the decline was steeper in the FMEA group, with the largest between-group difference at 24 h, and this advantage persisted through 72 h, suggesting that the FMEA-based nursing model provides both immediate and sustained analgesic effects. Concomitant with the improvement in pain scores, opioid consumption was also significantly reduced in the FMEA group. Repeated-measures ANOVA revealed a significant group-by-time interaction (*F* = 4.82, *p* = 0.003), with significant between-group main effect (*F* = 12.35, *p* < 0.001) and time main effect (*F* = 38.61, *p* < 0.001). Figure 3B shows that cumulative opioid use at 48 h was (28.1 ± 7.6) mg in the FMEA group versus (36.8 ± 9.4) mg in controls; at 72 h, values were (38.6 ± 9.3) mg and (50.4 ± 11.7) mg, respectively, representing a relative reduction of 23.4%. Table 2 further shows that the incidence of opioid-related adverse events was 6.4% in the FMEA group, significantly lower than the 12.6% in the control group. These findings indicate that FMEA-based bleeding risk-stratified nursing, through prospective bleeding risk precontrol, effectively reduces postoperative pain intensity in patients after coronary angiography while simultaneously decreasing opioid dependence and related adverse events, thereby breaking the passive “pain escalation – opioid dose increase” cycle and achieving synchronous improvements in analgesic efficiency and safety.

**Figure 3:**
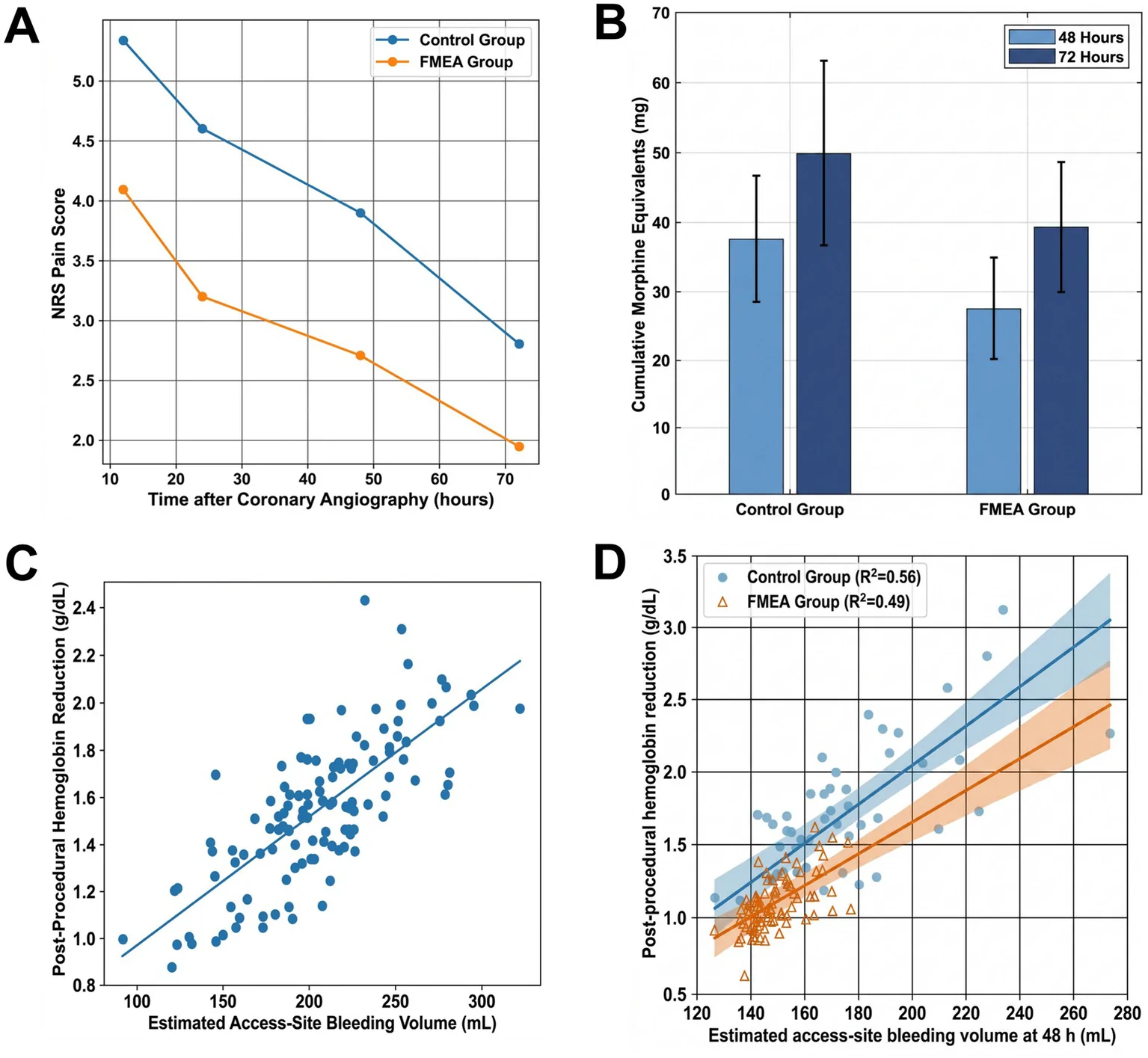
Comparison of postoperative pain control and access-site bleeding management outcomes between groups. **(A)** Trends in pain intensity (NRS scores) from 12 to 72 h postoperatively. **(B)** Comparison of cumulative opioid consumption at 48 h and 72 h postoperatively. **(C)** Correlation between postoperative haemoglobin decline and access-site bleeding volume. **(D)** Linear regression of estimated access-site bleeding volume versus postoperative haemoglobin decline in the two groups. This regression intercept comparison was a post-hoc exploratory analysis.

**Table 2:** Postoperative pain, analgesic consumption, and bleeding control outcomes after coronary angiography.

Variable	Control group (*n* = 120)	FMEA group (*n* = 120)	*p*-value
Pain intensity (NRS score)			
NRS pain score at 12 h	5.2 ± 1.2	4.1 ± 1.0	<0.001
NRS pain score at 24 h	4.6 ± 1.1	3.2 ± 0.8	<0.001
NRS pain score at 48 h	3.9 ± 0.9	2.7 ± 0.7	<0.001
NRS pain score at 72 h	2.8 ± 0.8	1.9 ± 0.6	<0.001
Analgesic consumption and adverse effects			
Cumulative opioid use at 48 h (mg, morphine equivalents)	36.8 ± 9.4	28.1 ± 7.6	<0.001
Cumulative opioid use at 72 h (mg, morphine equivalents)	50.4 ± 11.7	38.6 ± 9.3	<0.001
Opioid-related adverse effects (%)	12.6	6.4	0.048
Bleeding-related outcomes			
Clinically significant access-site bleeding (%)	17.5	8.3	0.031
Estimated access-site bleeding volume at 24 h (mL)	158.3 ± 44.9	121.6 ± 38.7	<0.001
Estimated access-site bleeding volume at 48 h (mL)	241.7 ± 60.8	186.4 ± 52.1	<0.001
Post-procedural hemoglobin reduction (g/dL)	2.1 ± 0.6	1.6 ± 0.4	<0.001
Requirement for hemostatic intervention at access site (%)	9.2	3.3	0.041
Unplanned access-site assessments (times/patient)	2.6 ± 0.9	1.7 ± 0.6	<0.001
Unplanned pain-related nursing calls (times/patient)	2.9 ± 1.1	1.8 ± 0.7	<0.001

Data source: Post-procedural nursing records, analgesic administration logs, and laboratory monitoring data following coronary angiography (January 2023–December 2024).

### 3.3 Access-site bleeding control and circulatory homeostasis maintenance

Access-site bleeding is the most common local complication after coronary angiography and a key initiating factor that exacerbates pain and delays recovery. Figure 3C shows the correlation between postoperative haemoglobin decline and estimated access-site bleeding volume; in both groups, estimated bleeding volume was positively associated with haemoglobin reduction, indicating that local bleeding is a major driver of postoperative haemoglobin decrease. Figure 3D further compares the linear regression relationships between estimated access-site bleeding volume and postoperative haemoglobin decline in the two groups; the intercept of the regression curve for the FMEA group was significantly lower than that for the control group, suggesting that, at similar bleeding volumes, haemoglobin levels were better preserved in the FMEA group. It should be noted that regression analyses were used as supplementary sensitivity analyses to adjust for potential confounders, and the primary conclusions were derived from direct between-group comparisons.

Table 2 summarises postoperative bleeding-related outcomes. The incidence of clinically significant access-site bleeding was 8.3% in the FMEA group, significantly lower than the 17.5% in the control group. Estimated bleeding volumes at 24 h were (121.6 ± 38.7) mL in the FMEA group versus (158.3 ± 44.9) mL in controls; at 48 h, values were (186.4 ± 52.1) mL and (241.7 ± 60.8) mL, respectively, representing a relative reduction of approximately 22.9%. Correspondingly, the postoperative haemoglobin decline was (1.6 ± 0.4) g/dL in the FMEA group, significantly less than the (2.1 ± 0.6) g/dL in controls. In addition, the proportion of patients requiring haemostatic intervention was 3.3% in the FMEA group versus 9.2% in controls, and unplanned access-site assessments were also significantly fewer: (1.7 ± 0.6) vs. (2.6 ± 0.9) times per patient. These results demonstrate that FMEA-based bleeding risk-stratified nursing, by prospectively identifying high-risk patients and matching them with differentiated compression haemostasis protocols and intensified monitoring, effectively controls access-site bleeding volume and its impact on circulatory homeostasis, thereby better preserving postoperative haemoglobin and providing a physiological foundation for early mobilisation and functional recovery.

### 3.4 Functional recovery and hospitalization efficiency improvement

Early functional recovery is a core indicator of postoperative rehabilitation quality. Figure 4A presents the Kaplan–Meier cumulative distribution curves for time to first ambulation; the curve for the FMEA group shifted markedly to the left, indicating that patients in the FMEA group achieved first ambulation earlier. At the 24-h time point, the estimated 24-h cumulative ambulation rate derived from the Kaplan–Meier curves was significantly higher in the FMEA group, and the median time to ambulation was 19.5 h in the FMEA group versus 27.9 h in controls. Regarding hospitalisation efficiency, the probability density distribution of postoperative length of stay showed a leftward shift in the FMEA group. Postoperative length of stay was significantly shorter in the FMEA group, and the proportion of stays exceeding 10 days was markedly lower than in controls (Figure 4B). Figure 4C shows that the incidence of severe bleeding (a severe subtype of clinically significant bleeding) was 7.5% in the FMEA group (vs. 17.5% in controls), prolonged hospitalisation (>10 days) occurred in 13.5% (vs. 29.0%), and the 30-day readmission rate was 5.8% (vs. 6.7%), with no significant difference in the latter.

**Figure 4:**
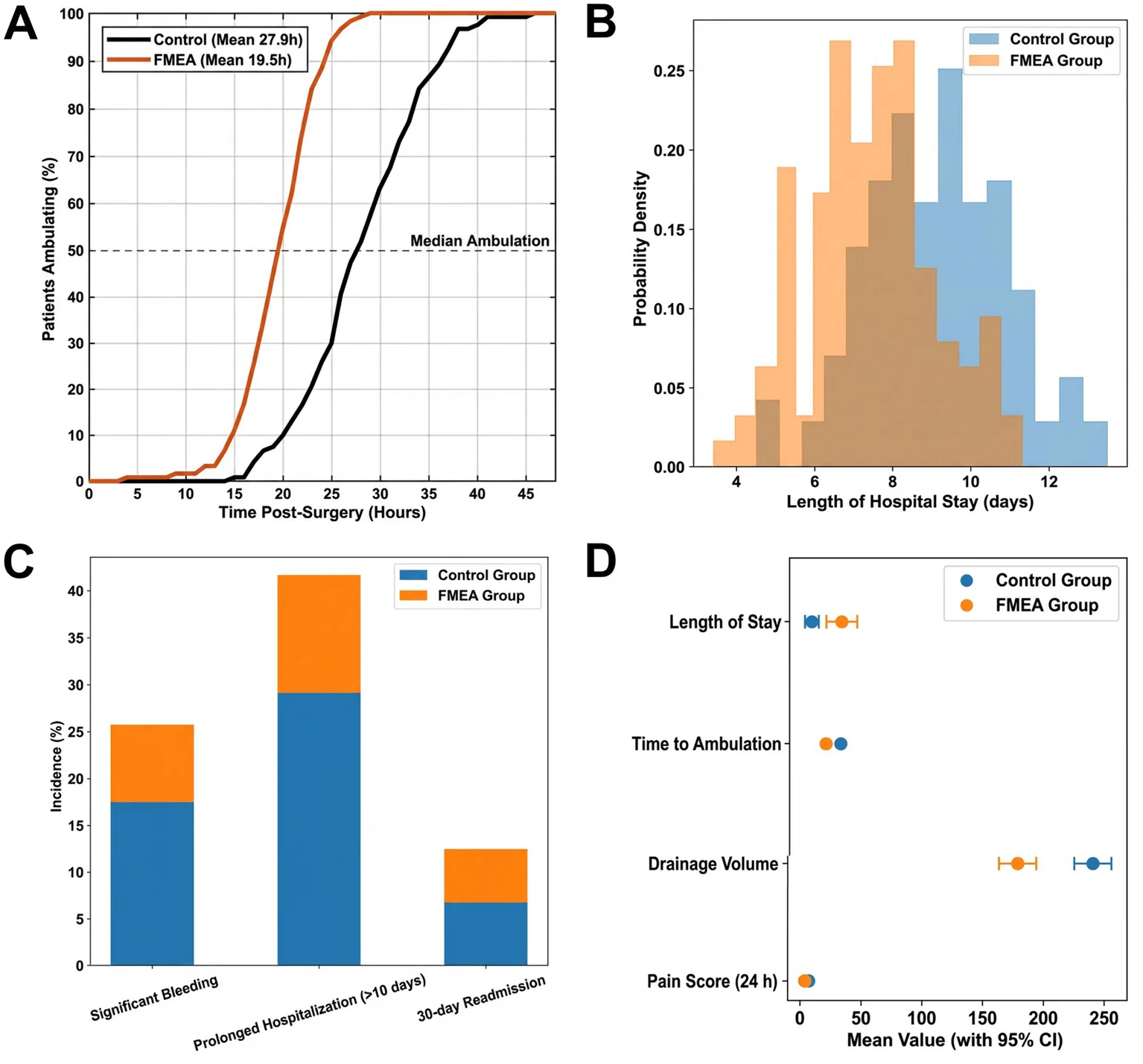
Comparison of postoperative functional recovery and hospitalisation efficiency outcomes between groups **(A)** Kaplan–Meier cumulative distribution curves for time to first ambulation. **(B)** Probability density distribution of postoperative length of hospital stay. **(C)** Comparison of adverse clinical outcomes (severe bleeding, prolonged hospitalisation, 30-day readmission) between groups. “Severe bleeding” denotes the subset of clinically significant bleeding requiring blood transfusion, surgical intervention, or hemodynamic instability. **(D)** Forest plot of standardised mean differences and 95% confidence intervals for key efficacy indicators.

Regarding discharge functional status, Table 3 shows that the FMEA group had significantly higher Functional Independence Measure (FIM) scores at discharge. Among subscores, improvements in mobility and transfer ability were most pronounced, whereas the self-care subscore did not differ significantly between groups. Additionally, patient-reported readiness for discharge scores were superior in the FMEA group; the proportion of patients referred to rehabilitation facilities after discharge was 28.3% in the FMEA group versus 41.7% in controls. Figure 4D presents a forest plot summarising the standardised mean differences and 95% confidence intervals for key indicators including pain scores, bleeding volume, ambulation time, and length of stay; all confidence intervals did not cross the line of no effect, further supporting the comprehensive advantages of FMEA-based nursing across multiple clinical dimensions. These results indicate that FMEA-based bleeding risk-stratified nursing, through effective control of access-site bleeding and postoperative pain, creates favourable conditions for early mobilisation, thereby accelerating functional recovery and shortening hospitalisation, without increasing short-term readmission risk, thus reflecting the harmonisation of rehabilitation efficiency and clinical safety.

**Table 3:** Post-coronary angiography rehabilitation recovery and hospitalisation outcomes.

Variable	Control group (*n* = 120)	FMEA group (n = 120)	*p*-value
Early mobilization recovery			
Time to first ambulation (h)	27.8 ± 6.1	19.6 ± 4.2	<0.001
Assisted ambulation within 24 h (%)	38.3	71.7	<0.001
Independent ambulation at discharge (%)	62.5	78.3	0.009
Time to stair climbing (days)	4.6 ± 1.3	3.4 ± 1.1	<0.001
Functional independence score at discharge			
Total score	74.1 ± 7.9	82.4 ± 6.7	<0.001
Mobility subscore	23.6 ± 4.1	27.8 ± 3.6	<0.001
Transfer ability subscore	18.9 ± 3.7	22.1 ± 3.4	<0.001
Self-care subscore	31.6 ± 4.8	32.5 ± 4.2	0.118
Hospitalization efficiency and safety			
Length of hospital stay after coronary angiography (days)	9.1 ± 2.0	7.3 ± 1.6	<0.001
Prolonged hospitalization (>10 days) (%)	29.2	12.5	0.002
Discharge delayed due to pain after coronary angiography (%)	18.3	7.5	—
Discharge delayed due to access-site issues (%)	14.2	6.7	—
30-day readmission rate after coronary angiography (%)	6.7	5.8	0.782
Patient-reported outcomes and transitional care			
Post-discharge rehabilitation referral (%)	41.7	28.3	0.032
Patient-reported readiness for discharge score	7.2 ± 1.1	8.4 ± 0.9	<0.001

Data source: Rehabilitation assessment records and hospital administrative databases following coronary angiography (January 2023–December 2024).

### 3.5 System-level safety and optimization of nursing resource allocation

System-level safety and resource utilisation efficiency are important dimensions for assessing the sustainability and generalisability of a nursing model. The radar chart (Figure 5A) shows that at discharge the FMEA group scored higher across all three FIM domains, mobility, transfer ability, and self-care, with a noticeably larger polygon area, and the between-group differences were especially prominent in mobility and transfer ability. A three-dimensional scatterplot (Figure 5B) illustrates the interplay among pain intensity, ambulation time, and length of stay; patients in the FMEA group clustered clearly in the region of low pain intensity, early ambulation, and short hospital stay, suggesting synergistic effects among bleeding control, pain relief, and functional recovery. The correlation matrix heatmap within the FMEA intervention group (Figure 5C) revealed that pain scores, bleeding volume, opioid use, ambulation time, and length of stay were positively correlated with each other, while all these variables were negatively correlated with functional scores, further validating the inter-relationships among recovery dimensions. Figure 5D shows that patients with less postoperative haemoglobin loss had higher discharge functional scores and shorter hospital stays, and patients in the FMEA group were more concentrated in the advantageous region of low blood loss and high function. The histogram of self-care function at discharge showed a rightward shift in the FMEA group, and patient-reported readiness-for-discharge scores confirmed better discharge preparedness in the FMEA group (Figures 5E,F).

**Figure 5:**
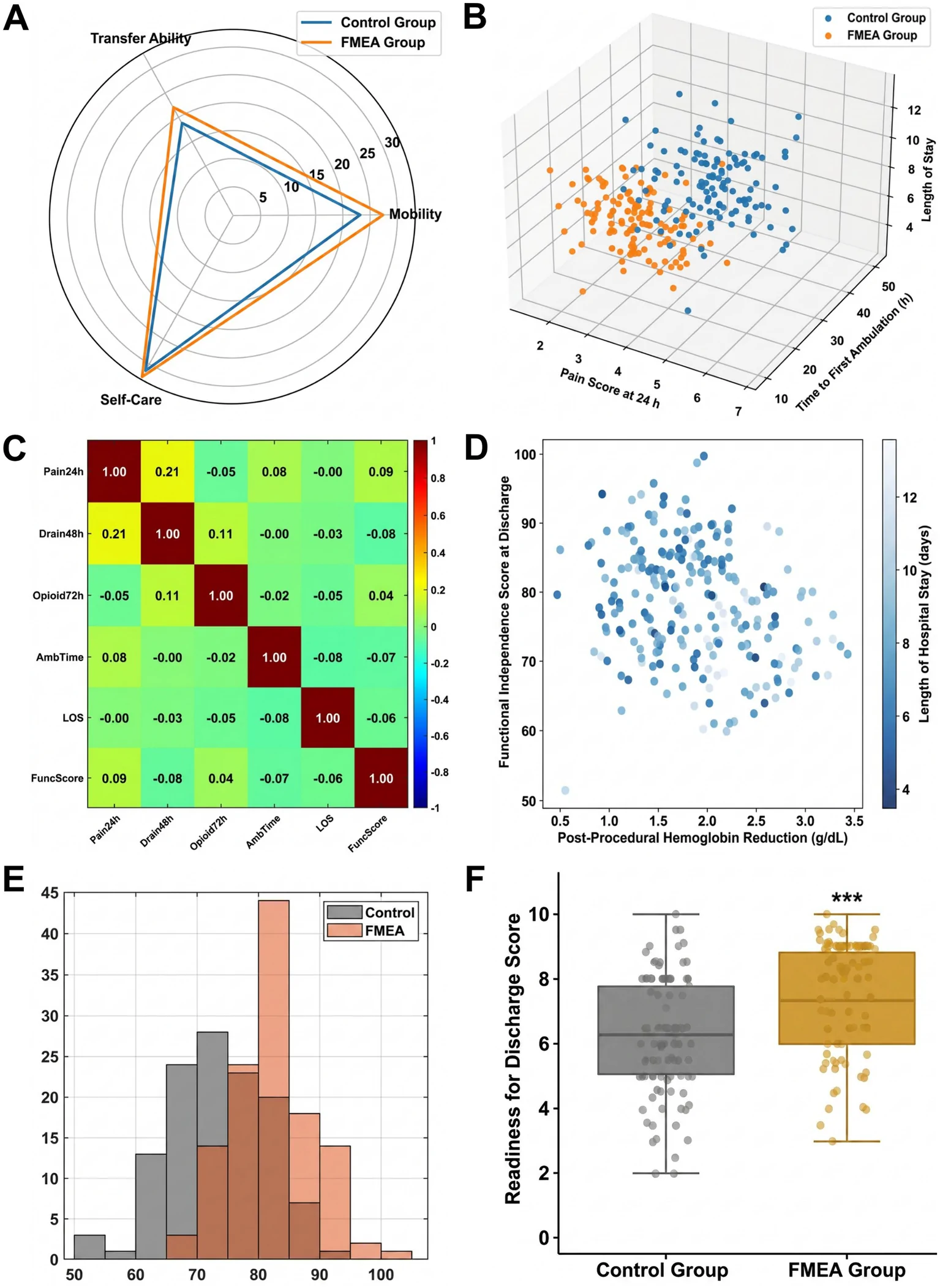
Comparison of multidimensional functional rehabilitation and system-level nursing resource utilisation between groups. **(A)** Radar chart of multidomain self-care function assessment at discharge. **(B)** Three-dimensional distribution of postoperative pain–ambulation time–length of stay recovery characteristics. **(C)** Correlation matrix heatmap of key recovery indicators within the FMEA intervention group. **(D)** Scatterplot of the interplay among postoperative haemoglobin loss, functional recovery, and length of stay. **(E)** Histogram of self-care function scores at discharge. **(F)** Comparison of patient-reported readiness-for-discharge scores.

Table 4 summarises system-level safety and resource utilisation indicators. Regarding safety, the incidence of clinically significant access-site bleeding was 8.3% in the FMEA group versus 17.5% in controls; opioid-related adverse events occurred in 6.4% vs. 12.6%; nausea and vomiting requiring intervention occurred in 10.8% vs. 18.3%; and postoperative hypotensive episodes occurred in 7.5% vs. 14.2%. Regarding resource utilisation, unplanned nursing interventions were (2.4 ± 0.9) times per patient in the FMEA group versus (3.8 ± 1.2) in controls; unplanned access-site assessments were (1.2 ± 0.5) vs. (1.9 ± 0.7); and emergency analgesic adjustments were required in 12.5% of FMEA patients versus 21.7% of controls. In addition, postoperative monitoring hours were shorter in the FMEA group; high-dependency monitoring utilisation was 16.7% versus 28.3%; night-shift escalation calls and nursing workload index were both significantly lower; and nursing documentation time was reduced. The safety incident reporting rate was also significantly lower in the FMEA group. These results indicate that FMEA-based bleeding risk-stratified nursing not only improves patient-level outcomes such as pain control, bleeding management, and functional recovery but also substantially reduces adverse event rates, unplanned nursing interventions, and resource consumption at the system level, thereby achieving synchronous optimisation of clinical safety and nursing efficiency, demonstrating the implications for efficiency of a “preventive care” approach over a “reactive care” model.

**Table 4:** System-level safety and resource utilisation outcomes after coronary angiography.

Indicator	Control group (*n* = 120)	FMEA group (*n* = 120)	*p*-value
Clinically significant access-site bleeding incidence (%)	17.5	8.3	0.031
Opioid-related adverse events (%)	12.6	6.4	0.048
Nausea and vomiting requiring intervention (%)	18.3	10.8	0.041
Post-procedural hypotension episodes (%)	14.2	7.5	0.037
Unplanned nursing interventions (times/patient)	3.8 ± 1.2	2.4 ± 0.9	<0.001
Unscheduled access-site assessments (times/patient)	1.9 ± 0.7	1.2 ± 0.5	<0.001
Emergency analgesic adjustments (%)	21.7	12.5	0.049
Post-procedural monitoring hours (h)	46.2 ± 8.7	38.9 ± 7.4	<0.001
High-dependency monitoring utilization (%)	28.3	16.7	0.018
Night-shift escalation calls (times/patient)	1.6 ± 0.6	0.9 ± 0.4	<0.001
Nursing workload index (score)	7.4 ± 1.3	6.1 ± 1.1	<0.001
Documentation time per patient (h)	5.8 ± 1.0	4.6 ± 0.9	<0.001
Safety incident reports (events/100 patients)	9.2	4.1	0.022

Data source: Nursing workload logs, postoperative safety reports, and institutional quality management records following coronary angiography (January 2023–December 2024). “Unplanned nursing interventions” denote unscheduled care activities triggered by patient condition changes or events; “nursing workload index” is a weighted score based on nursing hours per patient day (NHPPD); “safety incident reports” are postoperative safety events recorded in the hospital’s adverse event reporting system. All data were extracted by two trained researchers independently.

## 4 Discussion

The quality of recovery after coronary angiography has long been constrained by the vicious cycle between access-site bleeding and postoperative pain, while the traditional event-driven nursing model lacks prospective risk stratification and preemptive mechanisms to break this cycle at its source (23, 24). In this study, we introduced FMEA, a systems reliability analysis tool, into post-angiography nursing practice. Through structured failure mode identification and RPN-based quantitative scoring, we achieved individualised three-tier risk stratification and matched differentiated nursing intensities. In a prospective controlled design, we systematically evaluated the effects of this model on patient recovery trajectories and healthcare safety. Our results showed that the FMEA group was significantly superior to the control group across multiple dimensions, including postoperative pain control, opioid reduction, access-site bleeding management, accelerated functional recovery, improved hospitalisation efficiency, and system-level safety and resource utilisation optimisation. These findings suggest that FMEA-based bleeding risk-stratified nursing effectively interrupts the “bleeding–pain–immobilisation” cycle upstream and facilitates the transition from a reactive, event-driven model to a proactive, preemptive nursing paradigm.

We found that pain scores in the FMEA group were significantly lower than those in the control group at all time points, with this advantage persisting through 72 h, while cumulative opioid consumption and related adverse events were also substantially reduced. This pain management benefit is unlikely to be attributable solely to an intensified analgesic regimen; rather, it more likely originates from the upstream interruption of the “bleeding–inflammation–pain” vicious cycle enabled by the FMEA framework. By prospectively identifying high-bleeding-risk patients through preoperative RPN quantification, and matching them with stricter compression haemostasis protocols, more frequent access-site monitoring, and earlier recognition of bleeding signs, the FMEA framework curtails the triggering conditions before the local inflammatory cascade is initiated (25). This “upstream blocking” effect is indirectly corroborated by our data: estimated 48-h bleeding volume and postoperative haemoglobin decline were both significantly lower in the FMEA group. Reduced bleeding burden translates into a substantial diminution of inflammatory mediator release within local tissues, thereby lowering the input intensity of nociceptive signals; opioid demand consequently decreases naturally. Notably, the FMEA group achieved superior pain control with lower opioid consumption, a pattern that precisely reflects improved “analgesic efficiency,” rather than simply using more medication to suppress more severe pain. By attenuating the source drivers of pain, a limited dose of analgesics exerts a greater net effect (26, 27). This finding carries an important clinical implication: postoperative pain should not be narrowly interpreted as an isolated symptom requiring symptomatic pharmacological treatment; rather, it should be reconceptualised as a systemic signal reflecting local tissue homeostasis disruption. Effective pain management, therefore, cannot rely solely on pharmacological pathways, it also demands nursing risk precontrol to eliminate the source drivers of pain.

The improvements in pain and bleeding control, however, represent only the direct effects of the FMEA framework (28). Deeper changes occurred at the system level: the FMEA group had significantly fewer unplanned nursing interventions, night-shift escalation calls, emergency analgesic adjustments, and safety incident reports compared with the control group. The consistent improvement across these indicators suggests that the impact of the FMEA framework extends beyond optimising clinical outcomes to fundamentally reshape the underlying logic of nursing practice (17, 29). In the traditional nursing model, the nurse’s role is essentially that of a “stress responder,” initiating analgesic protocols after patients report pain, reinforcing compression after dressing oozing is detected, and reviewing safety measures after a fall occurs during ambulation. The problem with this model lies not in the technical correctness of nursing procedures per se, but in the fact that decision-making always lags behind the establishment of pathophysiological processes (30). The introduction of the FMEA framework alters this landscape: when a nurse identifies a patient as high-bleeding-risk based on RPN scores upon admission and initiates intensified monitoring and individualised compression protocols before any bleeding event has occurred, nursing behaviour shifts from “event-triggered” to “risk-triggered” (31). This transformation carries a dual significance for nursing professionalism. On one hand, it equips nurses with a data-driven clinical decision-making tool, RPN quantification standardises risk assessment from intuitive judgement to measurable metrics, providing an objective, auditable basis for nursing diagnoses. On the other hand, it shifts the focus of nursing work from “executing orders” to “identifying risks”; nurses are no longer merely executors of analgesic prescriptions but become early warning sentinels and proactive interveners in the patient’s recovery process. This role transition aligns closely with the contemporary evolution of the nursing discipline from a “task-oriented” to an “outcome-oriented” paradigm (32). From a potential resource utilization benefits, the FMEA framework precisely aligns nursing resource allocation with individual patient risk levels, thereby avoiding the structural inefficiency that characterises conventional models, where high-risk patients receive insufficient monitoring while low-risk patients receive superfluous surveillance. The concurrent reductions in postoperative monitoring hours, high-dependency monitoring utilisation, and nursing documentation time collectively indicate that preemptive nursing achieves outcome improvement not at the cost of increased resource consumption, but by optimising the forward-looking allocation of resources, thereby reducing system operating burdens while lowering safety incident rates (33). In the context of the ongoing transition of healthcare payment systems from fee-for-service to value-based models such as DRG/DIP, this model is expected to become a critical pathway for synergistically enhancing the quality and cost-effectiveness of interventional nursing care. It is important to note that reductions in monitoring time, documentation time, and unplanned nursing interventions should be interpreted as indicators of process efficiency and structured workflow optimisation, rather than as direct evidence of improved safety. The primary evidence for safety improvement comes from the clinical outcomes, reduced bleeding events, less haemoglobin decline, and lower opioid consumption, which were consistently favourable in the FMEA group.

Among the most clinically translatable findings of this study is the significant reduction in length of hospital stay in the FMEA group, accompanied by a marked decline in the proportion of prolonged hospitalisations (34). More critically, the 30-day readmission rates did not differ significantly between groups, indicating that the shortened hospital stay was not achieved at the expense of increased post-discharge adverse event risk, but rather represented a genuine efficiency gain grounded in true functional improvement. The FMEA group also demonstrated significantly higher Functional Independence Measure scores and patient-reported readiness-for-discharge scores, further confirming that patients in the FMEA group had better self-care capacity and functional reserve at the time of discharge (35). Viewed through the lens of “recovery process,” the FMEA framework effectively altered the entire shape of the postoperative recovery curve: effective upfront bleeding risk control reduced the area under the pain curve; attenuation of pain drivers advanced the timing of first ambulation; early mobilisation facilitated faster recovery of muscle strength and joint function; and this ultimately translated into shortened hospital stays and enhanced discharge functional status, constituting a consistent pattern of associations, rather than a simple superposition of isolated clinical events (36). Notably, the proportion of patients referred to rehabilitation facilities after discharge was significantly lower in the FMEA group, suggesting that the recovery quality in the FMEA group had reached a higher “discharge-to-independence” threshold, reducing dependence on post-discharge continuing rehabilitation resources. This finding has implications for the design of post-discharge management pathways for interventional patients within the context of tiered healthcare delivery.

Several limitations of this study should be acknowledged. First, this was a single-centre study conducted at one tertiary teaching hospital; the nursing team composition, FMEA implementation experience, and patient case mix may differ from those at other institutions, and the generalisability of our findings should be validated through multicentre studies. Second, due to the nature of the nursing intervention, blinding of nursing staff and patients was not feasible, which may introduce certain measurement and performance biases, although we used objective outcome measures wherever possible to minimise the influence of subjective judgement on results. Third, follow-up was limited to 30 days postoperatively, focusing primarily on in-hospital recovery trajectories and short-term safety outcomes; the long-term effects of the FMEA-based nursing model on patients’ quality of life, cardiovascular events, and sustained functional maintenance remain unclear. Future studies should extend the follow-up period to evaluate the longer-term impact of this model. Fourth, although we achieved systematic risk stratification and differentiated intervention at the nursing level, intraoperative variables were not incorporated into the stratification model; the interactions of these factors with postoperative bleeding risk warrant further exploration. Subsequent studies may incorporate intraoperative parameters such as puncture attempts, vascular anatomical variations, and operator experience into the RPN model to build a more comprehensive dynamic bleeding risk prediction system. Fifth, nursing complexity and individual patient care demands may independently influence length of stay, functional recovery, and nursing workload, thereby potentially confounding the net effect of risk-stratified interventions (19, 21, 22, 37–41). Future studies should integrate standardized nursing-generated data and measures of nursing complexity to better understand their relationship with clinical and organizational outcomes, and to disentangle the contribution of underlying care complexity from the effects of risk-stratified nursing interventions. Sixth, although we used structured indicators to estimate nursing workload and resource utilisation, we acknowledge that some key nursing activities may remain embedded in unstructured free-text documentation and thus be overlooked in our current assessment (42). Future studies should also consider integrating structured nursing indicators with information extracted from free-text clinical documentation, as natural language processing approaches may capture nursing activities that are otherwise not represented in structured data and provide a more comprehensive assessment of nursing care delivery and resource utilization. We also acknowledge the potential “Hawthorne effect,” the possibility that patients in the intervention group may have improved simply because they received more attention from the nursing team. Although this is an inherent limitation of behavioural intervention studies, we sought to minimise its influence through standardised assessment protocols, cross-validation of objective and subjective outcomes, and blinded outcome assessment. Nonetheless, this effect cannot be completely ruled out and should be considered when interpreting the findings.

## 5 Conclusion

This single-centre prospective controlled study suggests that FMEA-based bleeding risk-stratified nursing was associated with improvements in short-term bleeding-related outcomes, pain-related outcomes, mobility recovery, and selected workflow efficiency indicators. These findings support the potential of the FMEA framework to facilitate the transition from an event-driven, reactive nursing model to a risk-driven, preemptive approach. However, given the exploratory nature of this study, the single-centre setting, non-blinded design, and short-term follow-up, the results should be interpreted as preliminary, and the causal relationships inferred from the “bleeding–inflammation–pain” framework require further validation through multicentre studies with extended follow-up and direct biomarker measurements.

## Data Availability

The original contributions presented in the study are included in the article/supplementary material, further inquiries can be directed to the corresponding author.
